# Diabetic retinopathy risk prediction in patients with type 2 diabetes mellitus using a nomogram model

**DOI:** 10.3389/fendo.2022.993423

**Published:** 2022-11-16

**Authors:** Qian Wang, Ni Zeng, Hongbo Tang, Xiaoxia Yang, Qu Yao, Lin Zhang, Han Zhang, Ying Zhang, Xiaomei Nie, Xin Liao, Feng Jiang

**Affiliations:** ^1^ Department of Endocrinology, Affiliated Hospital of Zunyi Medical University, Zunyi, China; ^2^ Department of Dermatology, Affiliated Hospital of Zunyi Medical University, Zunyi, China; ^3^ Department of Critical Care Medicine, The Third Affiliated Hospital of Zunyi Medical University (The First People’s Hospital of Zunyi), Zunyi, China; ^4^ Department of Integrated (Geriatric) Ward, The Second Affiliated Hospital of Zunyi Medical University, Zunyi, China; ^5^ Department of Cardiology, Affiliated Hospital of Zunyi Medical University, Zunyi, China; ^6^ Department of Ophthalmology, The Second Affiliated Hospital of Zunyi Medical University, Zunyi, China; ^7^ Department of Neonatology, Obstetrics and Gynecology Hospital of Fudan University, Shanghai, China

**Keywords:** nomogram model, type 2 diabetes mellitus, diabetic retinopathy, risk, prediction

## Abstract

**Background:**

This study aims to develop a diabetic retinopathy (DR) hazard nomogram for a Chinese population of patients with type 2 diabetes mellitus (T2DM).

**Methods:**

We constructed a nomogram model by including data from 213 patients with T2DM between January 2019 and May 2021 in the Affiliated Hospital of Zunyi Medical University. We used basic statistics and biochemical indicator tests to assess the risk of DR in patients with T2DM. The patient data were used to evaluate the DR risk using R software and a least absolute shrinkage and selection operator (LASSO) predictive model. Using multivariable Cox regression, we examined the risk factors of DR to reduce the LASSO penalty. The validation model, decision curve analysis, and C-index were tested on the calibration plot. The bootstrapping methodology was used to internally validate the accuracy of the nomogram.

**Results:**

The LASSO algorithm identified the following eight predictive variables from the 16 independent variables: disease duration, body mass index (BMI), fasting blood glucose (FPG), glycated hemoglobin (HbA1c), homeostatic model assessment-insulin resistance (HOMA-IR), triglyceride (TG), total cholesterol (TC), and vitamin D (VitD)-T3. The C-index was 0.848 (95% CI: 0.798–0.898), indicating the accuracy of the model. In the interval validation, high scores (0.816) are possible from an analysis of a DR nomogram’s decision curve to predict DR.

**Conclusion:**

We developed a non-parametric technique to predict the risk of DR based on disease duration, BMI, FPG, HbA1c, HOMA-IR, TG, TC, and VitD.

## Introduction

Currently, diabetes mellitus (DM) is one of the fastest-growing chronic diseases worldwide. An epidemiological study conducted between 2015 and 2017 in 31 cities and provinces of China indicated that the prevalence of DM was 11.0% (1). According to the International Diabetes Federation, there were 463 million diabetic patients between 20 and 79 years in 2019 globally. This number is expected to reach 578 million by 2045 and 700 million by 2045 (2). High blood sugar is a crucial marker of DM that damages the body’s microvasculature. For instance, diabetic retinopathy (DR) is a type of microangiopathy caused by diabetes. It is becoming increasingly common, as the number of people with DM is rising. By 2030, the estimated number of non-proliferative DR and progressive DR would be 191 million and 56.3 million, respectively. Additionally, due to a lack of awareness and understanding of DR, it has become the leading cause of vision loss in adults aged 30–60 years, significantly affecting the quality of life and posing a health risk. DR is silent at its inception; hence, its early detection, prevention, and therapy are critical for lowering its effect on life and social resources.

Several factors influence the onset and progression of DR. Hyperglycemia, hypertension, dyslipidemia, obesity, smoking, anemia, a lack of health information, and poor treatment adherence are risk factors of DR that can be altered. Whereas ethnicity, family history or inheritance, diabetes onset age, type of diabetes, and diabetes duration are all constant risk factors (3). Previously, studies have built a predictive model for the risk of DR in patients with type 2 diabetes mellitus (T2DM); however, the independent variables were different in all studies (4–6). Thus, this study collected the independent variables that have not appeared in previous studies and aimed to develop a comprehensive model for predicting the risk of DR and the need for early intervention.

## Methods

### Patients

We included 213 inpatients and outpatients with T2DM at the Affiliated Hospital of Zunyi Medical University between January 2019 and May 2021. The clinical data were collected in Zunyi, Guizhou, a southwestern region of China; hence, the participants were all Han Chinese. All patients with diabetes satisfied the WHO diagnostic criteria, including a fasting plasma glucose level >126 mg/dl and/or an oral glucose level of 75 mg 2 h later. Blood glucose was >200 mg/dl (7). The exclusion criteria were as follows: 1) People with T1DM and other forms of diabetes, including Cushing syndrome; 2) Patients with diabetes with acute diabetic complications, such as ketoacidosis; 3) Pregnant and lactating women; 4) Patients who were unable to perform a fundus examination, such as those with severe refraction in eyes and myopia/hyperopia with a history of >3 days; 5) Patients with eye diseases, such as glaucoma and severe cataracts, that affect fundus observation; 6) Patients with any other disease that can cause fundus hemorrhage; 7) Patients who consumed drugs that affect lipid metabolism and vitamin D (VitD) in the last 6 months. All individuals with T2DM were screened using CR-PGi (Canon). The CR-PGi captured no-dilatation fundus photographs, which have better sensitivity and specificity for screening DR, and high-quality fundus photographs can screen out the most clinically significant DRs (8). Patients screened for suspected DR were then referred to the ophthalmology department and underwent no-dilatation fundus photography, fluorescein fundus angiography, and optical coherence tomography by the same ophthalmologist to verify the diagnosis of DR. Data on gender, age, disease duration, systolic blood pressure (SBP), diastolic blood pressure (DBP), body mass index (BMI), fasting blood glucose (FPG), glycated hemoglobin (HbA1c), homeostatic model assessment-insulin resistance (HOMA-IR), triglyceride (TG), total cholesterol (TC), low-density lipoprotein cholesterol (LDL-C), high-density lipoprotein cholesterol (HDL-C), VitD, and creatinine (Cr) were obtained for all individuals *via* medical records. HOMA-IR was used to assess insulin resistance [IR index = FPG (mmol/L) × FINS (mU/L)/22.5] ≥2.8 (9, 10).

### Statistical analysis

Using least absolute shrinkage and selection operator (LASSO) regression, we improved the stability of the model by fitting a generalized linear model and performing variable selection and complexity adjustment (regularization). It screens the statistically significant independent variables and calculates the dominance ratio [odds ratios (OR)], 95% confidence interval (CI), and P-value for each independent variable. Finally, we performed a multifactor logistic regression analysis on the statistically significant independent variables (5). The proposed methodology was then built using heterogeneous logistic regression and properties of the cable regression model. Characteristics also included frequency or P-value with 95% CI. We decided to employ bidirectional statistically significant results to construct a predictive nomogram. A P-value <0.05 was considered statistically significant. To forecast the DR incidence, we constructed a prediction model with appropriate adjustments and graphical representations. The accuracy of the DR nomogram was evaluated using the C-index (11, 12), which was validated through bootstrapping. Preference curve analysis was used to determine the predicted results using a copy based on the projected benefit at scene probabilities (6).

## Results

### Characteristics of the patients

In total, 213 patients with T2DM, including 59.15% men and 40.85% women were included. The patients’ mean age was 55.25 ± 9.34 years (range 29–83 years). Based on the fundus examination, the patients were divided into two groups: non-DR (53.05%) and DR (46.95%). **Table 1** illustrates the essential characteristics of patients, including the detailed information of 16 clinical indicators.

**Table 1:** Differences between demographic and clinical characteristics of the non-DR and DR groups.

Demographic characteristics	Non-DR (n = 113)	DR (n = 100)	Total (N = 213)
Age (years)			
<50 50–70 >70	4 (44.44) 107 (55.15) 2 (0.20)	5 (55.56) 87 (44.85) 8 (0.80)	9 (4.23) 194 (91.08) 10 (4.69)
Gender			
Male Female	68 (53.97) 45 (51.72)	58 (46.03) 42 (48.28)	126 (59.15) 87 (40.85)
Disease duration (years)			
<5 5 < 10 ≥10	65 (76.47) 46 (40.00) 2 (15.38)	20 (23.53) 69 (60.00) 11 (84.62)	85 (39.91) 115 (53.99) 13 (6.10)
BMI			
<24 24 < 28 ≥28	66 (72.53) 37 (38.14) 10 (40.00)	25 (27.47) 60 (61.86) 15 (60.00)	91 (42.72) 97 (45.54) 25 (11.74)
SBP (mmHg)			
<140 ≥140	102 (52.04) 11 (64.71)	94 (47.96) 6 (35.29)	196 (92.02) 17 (7.98)
DBP (mmHg)			
<90 ≥90	99 (51.83) 14 (63.64)	92 (48.17) 8 (36.36)	191 (89.67) 22 (10.33)
Clinical characteristics			
FPG (mmol/L)			
<7.0 ≥7.0	41 (95.35) 72 (42.35)	2 (4.65) 98 (57.65)	43 (20.19) 170 (79.81)
HbA1c (%)			
<7 ≥7	34 (87.18) 79 (45.40)	5 (12.82) 95 (54.60)	39 (18.32) 174 (81.69)
FINs (mU/L)			
<5 5 < 20 ≥20	11 (78.57) 98 (50.78) 4 (66.67)	3 (21.43) 95 (49.22) 2 (33.33)	14 (6.57) 193 (90.61) 6 (2.82)
HOMA-IR			
<2.8 ≥2.8	33 (80.49) 80 (46.51)	8 (19.51) 92 (53.49)	41 (19.25) 172 (80.75)
TG (mmol/L)			
<1.7 ≥1.7	48 (66.67) 65 (46.10)	24 (33.33) 76 (53.90)	72 (33.80) 141 (66.20)
TC (mmol/L)			
<5.2 ≥5.2	69 (53.91) 44 (51.76)	59 (46.09) 41 (48.24)	128 (60.09) 85 (39.91)
HDL-C (mmol/L)			
>1.0 ≤1.0	45 (50.56) 68 (54.84)	44 (49.44) 56 (45.16)	89 (41.78) 124 (58.22)
LDL-C (mmol/L)			
<2.6 ≥2.6	42 (58.33) 71 (50.35)	30 (41.67) 70 (49.65)	72 (33.80) 141 (66.20)
VitD (ng/ml)			
≥30 15 < 30 <15	4 (80.00) 81 (62.31) 28 (35.90)	1 (20.00) 49 (37.69) 50 (64.10)	5 (2.35) 130 (61.03) 78 (36.62)
Cr (µmol/L)			
≥90 <90	104 (55.03) 9 (37.50)	85 (44.97) 15 (52.50)	189 (88.73) 24 (11.27)

BMI, body mass index; Cr, creatinine; DBP, diastolic blood pressure; DR, diabetic retinopathy; FPG, fasting blood glucose; HbA1c, glycated hemoglobin; HDL-C, high-density lipoprotein cholesterol; HOMA-IR, homeostatic model assessment-insulin resistance; LDL-C, low-density lipoprotein cholesterol; SBP, systolic blood pressure; TC, total cholesterol; TG, triglyceride; VitD, vitamin D.

### Variables to be chosen

**Figure 1:**
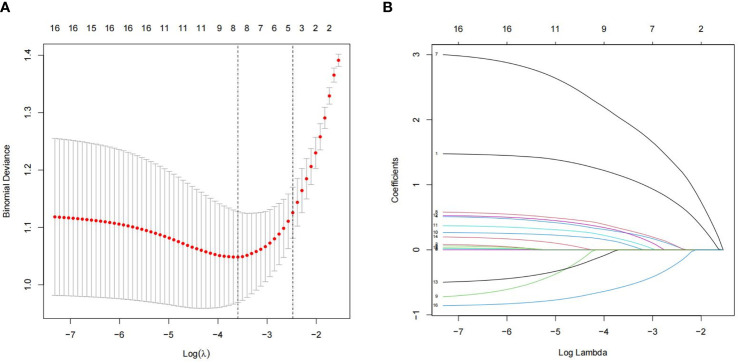
Least absolute shrinkage and selection operator (LASSO) logistic regression for assessing the relationship between populations and clinical features. **(A)** The optimum parameter in the LASSO regression model was chosen using a threshold of 16 cross-validations. The partial probability variance curves and the logarithm turn are shown. To get the best estimate, we used the 1-SE technique with minimum standards. **(B)** For each of the 16 characteristics, the LASSO logistic regression coefficients were calculated. The logarithmic lambda was used to determine the profile. The ideal lambda was tested using a cross-validation technique, with the optimal lambda producing eight coefficients.

We analyzed 16 independent variables using R software. Among these, eight statistically significant independent variables or lambda coefficients, namely, disease duration, BMI, FPG, HbA1c, HOMA-IR, TG, TC, and VitD, were screened using LASSO regression analysis (**Figures 1A, B**).

### Model development for diabetic retinopathy prediction

**Figure 2:**
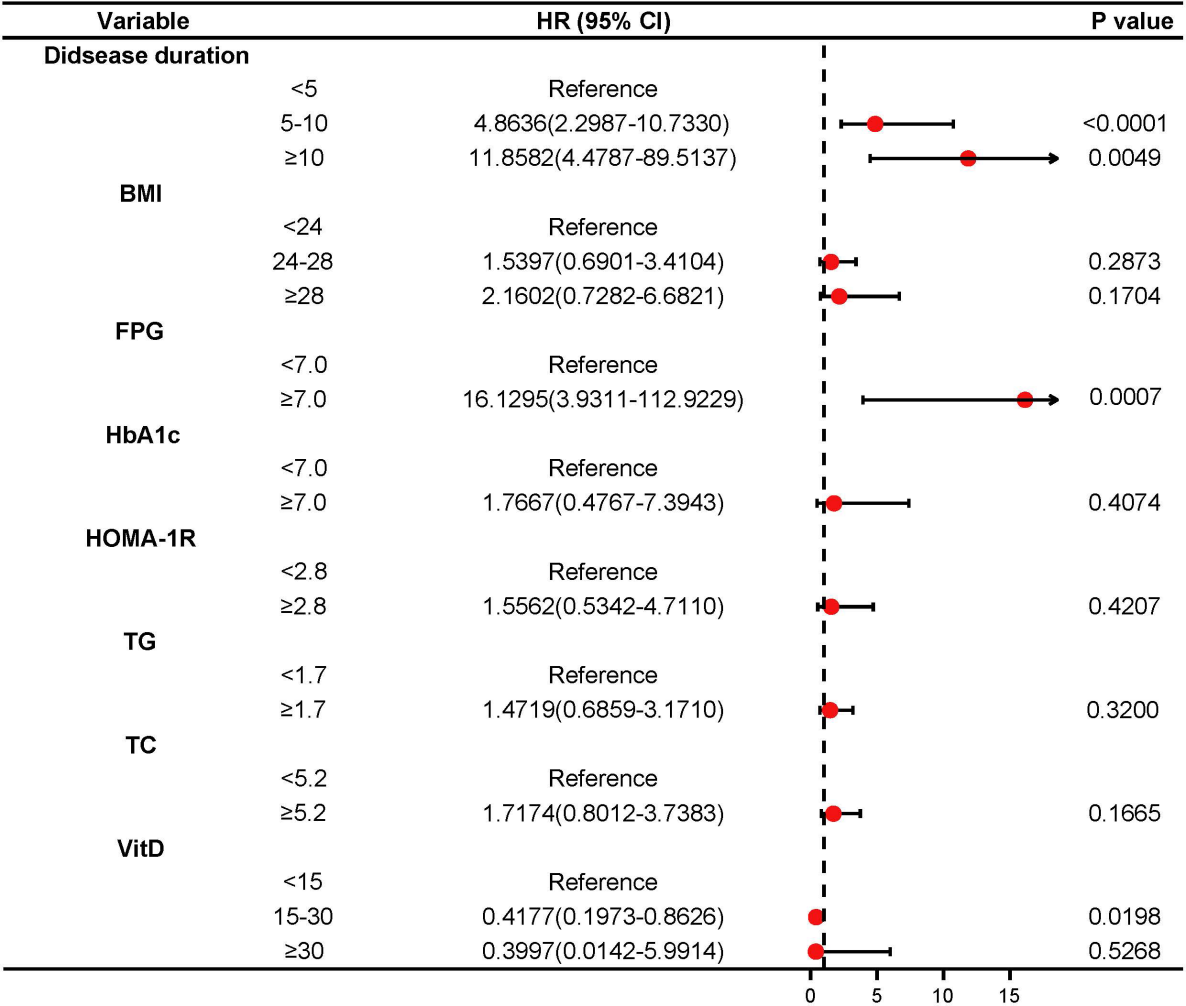
The forest plot of the odds ratio (OR) of the Cox regression results.

**Figure 3:**
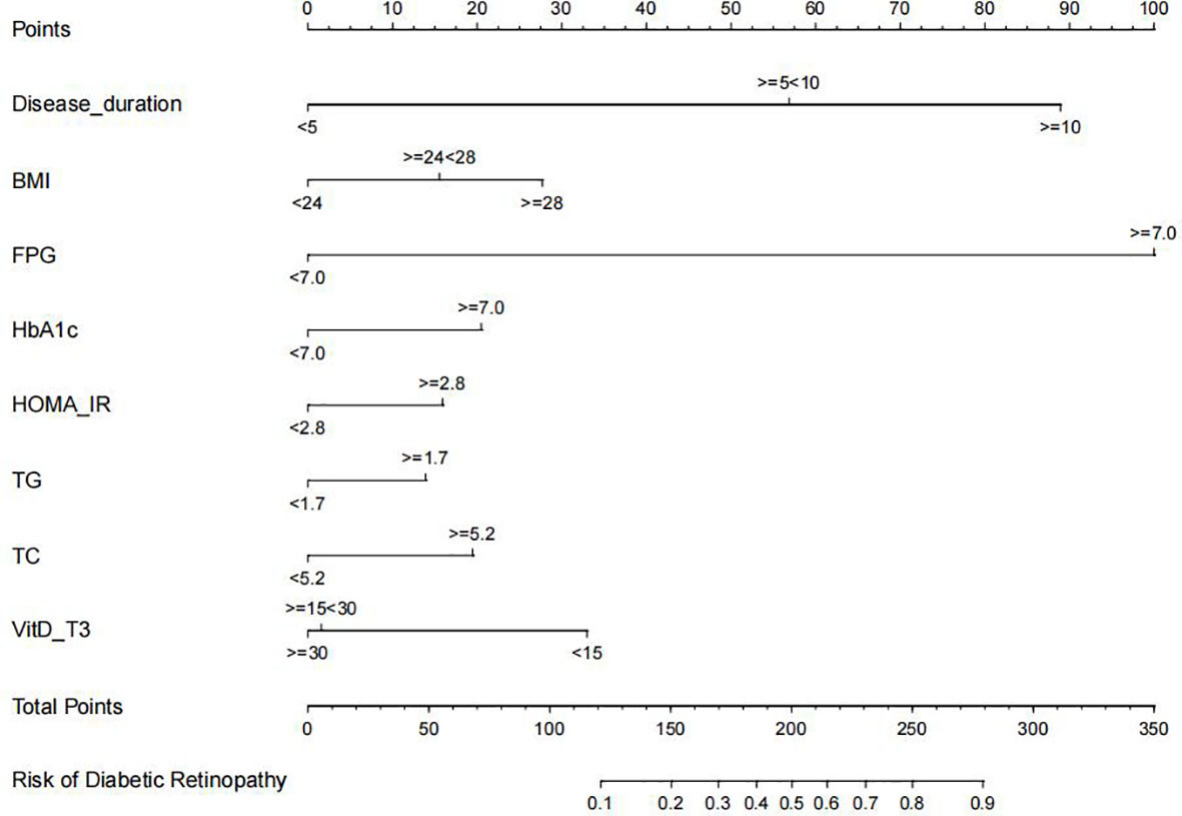
Diabetic retinopathy (DR) nomogram. The graph was created using data from the following sources: disease duration, body mass index (BMI), fasting blood glucose (FPG), glycated hemoglobin (Hb1Ac), homeostatic model assessment-insulin resistance (HOMA-IR), triglyceride (TG), total cholesterol (TC), and vitamin D (VitD)-T3.

**Figure 4:**
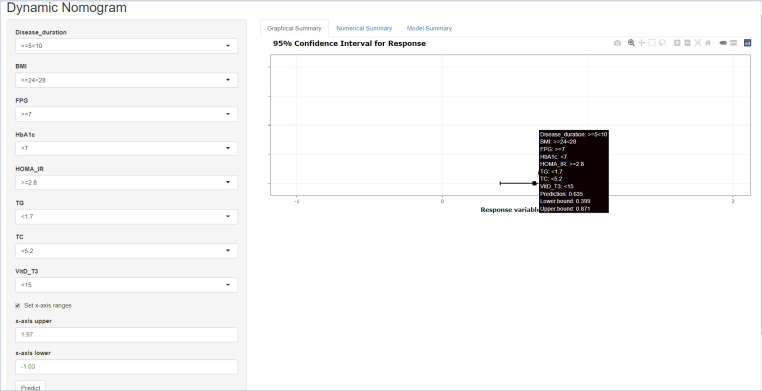
A web-based dynamic calculator for predicting the diabetic retinopathy (DR) risk with a 95% confidence interval.

Furthermore, disease duration, BMI, FPG, HbA1c, HOMA-IR, TG, TC, and VitD were examined using multivariate logistic regression analysis. The results of the Cox regression analysis were presented as forest plots (**Figure 2**). We obtained the following as the DR risk factors: Disease duration (5 < 10, OR = 4.8636; ≥10, OR = 11.8582), BMI (24 < 28, OR = 1.5497; ≥28, OR = 2.1602), FPG (≥7.0, OR = 16.1295), HbA1c (≥7.0, OR = 1.7667), HOMA-IR (≥2.8, OR = 1.5562), TG (≥1.7, OR = 1.4719), TC (≥5.2, OR = 1.7174), and VitD (15 < 30, OR = 0.4177; ≥30, OR = 0.3997). Using the aforementioned independent predictors, we created a model and presented it using a nomogram (**Figure 3**). Finally, we created a dynamic web-based calculator (https://dxyjiang.shinyapps.io/DRprediction/) that calculates the total score from each patient’s clinical indicators for determining the risk of developing DR (**Figure 4**).

### Accuracy of the cohort diabetic retinopathy exposure nomogram

**Figure 5:**
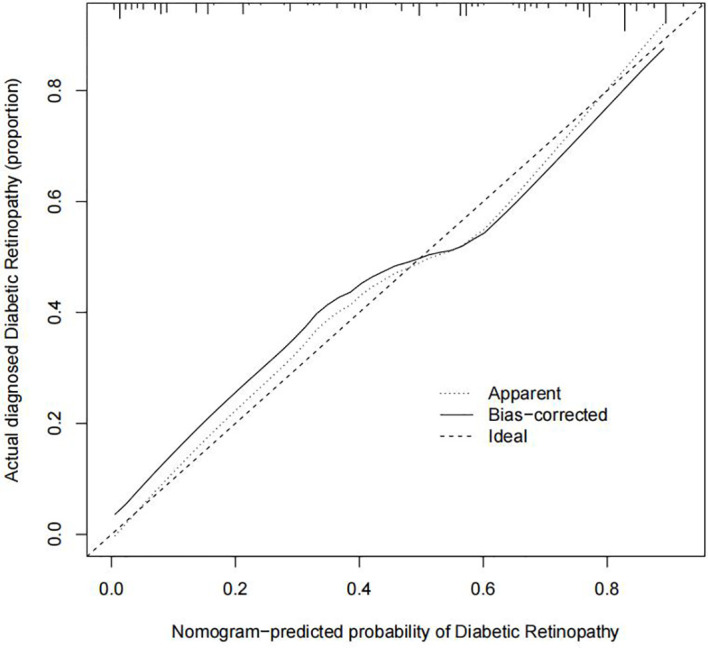
Prediction calibration curves of the diabetic retinopathy (DR) nomogram. The x-axis represents the possibility for DR. The x-axis reflects the nomogram-predicted probability. The y-axis reflects the actual predicted probability. A perfect prediction model describes an ideal forecast. The graph depicts the fitness of the nomogram for forecasting outcomes, the dotted line indicating a more reliable prediction.

The C-index for evaluating the occurrence of DR in patients with T2DM was 0.848 (95% CI: 0.798–0.898), demonstrating a high validity (**Figure 5**). Additionally, the result of the bootstrap verification was 0.816. These results indicate that the model has good prediction accuracy.

### Clinic application

**Figure 6:**
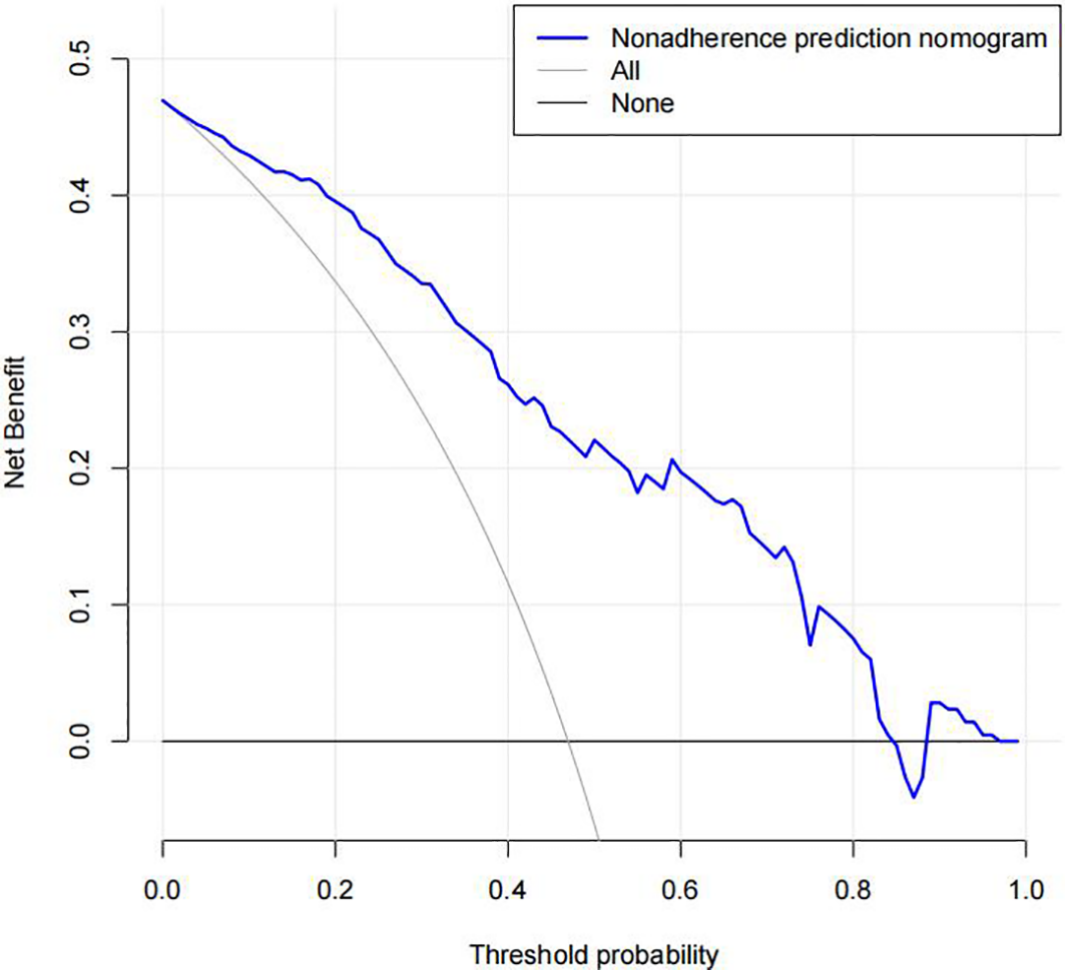
Decision curve analysis of the diabetic retinopathy (DR) nomogram. The x-axis represents the threshold probability. The y-axis represents the net benefit. The dotted line represents a DR risk nomogram, and the narrow solid line indicates patients presumed to have DR. The decision curve revealed that if a patient’s and a doctor’s threshold probabilities are more than 2% and 85%, respectively, using this DR nomogram in the current study to predict DR incidence risk adds more benefit than the intervention-all-patients scheme.

The DR nomogram was made up of scales for several variables to calculate the likelihood of a given result. According to the judgment curve, non-adherence use of the nomogram raises the projected chance of DR incidence if the thresholds of the patient and doctor are >2% and 85%, respectively. The overlaps were compiled in this study to ensure an equal net gain (**Figure 6**).

## Discussion

Nomograms are simple, quick, cheap, and noninvasive techniques to monitor patients and make appropriate clinical treatment decisions (13). They are used in various medical professions, such as for predicting tumor diagnostic outcomes and therapeutic effects. Nonetheless, only a few studies have attempted to forecast the risk of DR in patients with T2DM. Hence, this study gathered clinical data and demographic characteristics of patients with T2DM and constructed a new prediction model for determining the probability of acquiring DR.

Currently, due to high sensitivity and specificity, fundus photography is routinely employed in clinical settings for DR screening (8, 14). Additionally, screening merely evaluates the outcomes and does not reveal the components that play a part in creating the impact. DR is known to be one of the long-term effects of T2DM. It is widely accepted that diabetes is a potential risk for DR; nonetheless, it is unclear whether other factors lead to the development of DR. What is the significance of the relationship between DR and factors? Interestingly, nomograms can provide answers to all of these questions.

According to studies such as the Diabetes Control and Complications Trial (DCCT) and the UK Prospective Diabetes Study (UKPDS), overweight, disease duration, hypoglycemia, hypertension, high cholesterol, kidney disease, renal failure [diabetic kidney disease (DKD)], pregnancy, and susceptibility genes are common triggers for DR (15, 16). In this study, gender, age, disease duration, SBP, DBP, BMI, FPG, HbA1c, TG, TC, HOMA-IR, LDL-C, HDL-C, VitD-T3, and Cr, which are clinically available clinical indicators, were correlated with the risk of DR using the LASSO method. Furthermore, multivariate logistic regression analysis identified disease duration, BMI, FPG, HbA1c, HOMA-IR, TG, TC, and VitD as risk factors for DR. Except for disease duration, all other risk factors are modifiable. In short, the value of our model is the identification and management of such modifiable risk factors.

Recently, hyperglycemia and disease duration have been identified as risk factors for the pathogenesis of DR (17, 18). Consistently, our prediction model suggests greater risk levels for high fasting glucose and disease progression. Moreover, DR is a metabolic disorder that is difficult to treat and does not develop in patients with reasonable glycemic control. In contrast, patients with poor glycemic control are more likely to develop DR, implying that there are additional secondary contributing factors. According to previous studies, the probability of developing DR increases by approximately 64% per 10% increase in HbA1c and the two have a positive relationship (19–22). Lower serum levels may decrease the risk of severe blindness by 47% when compared with normal serum levels after approximately 20 years of follow-up (23). In addition, lipotoxicity, damage to the retinal barrier caused by excessive blood lipids, and exceptionally high TGs are a vital part of DR (24, 25). Thus, controlling dyslipidemia in addition to glycemic management is critical for preventing and treating DR (26). According to the independent variables assessed in our prediction model, HbA1c and lipids could be risk factors for DR. Thus, lowering blood glucose and controlling lipids are among the most critical preventive and therapeutic strategies for DR.

Although nomogram models have been used earlier to predict the risk of DR (6), our model yielded a higher C-index value, indicating a higher accuracy. The indicators included in our model are more comprehensive than those included in the previous models, making it a more accurate predictor of risk. This study included popular Vitamin D (VD) from recent years that other researchers have not used. Low levels of VD are a specific and sensitive sign of proliferative diseases. Additionally, they are adversely associated with the intensity of DR. The AUC recommends VD as a straightforward, sensitive, and specific laboratory test for DR (27). According to a foreign cross-sectional investigation, patients with VD insufficiency were more likely to acquire DR than those with VD sufficiency. Multidisciplinary ordinal regression analysis revealed a link between VD shortage and DR severity (28–30). Consistently, this study identified VD rates as a predictor of DR, and the incidence of DR increases with decreasing VD levels. Relevant mechanisms have been proposed for determining the role of VD in DR, including VD increasing endothelial nitric oxide synthase (eNOS)-dependent NO production, reducing oxidative stress, and enhancing pathophysiological processes, such as vascular endothelial growth factor (VEGF) synthesis and release (31–33). However, according to a prospective observational study, VD deficit is directly linked to all-cause survival and does not predict the development of microvascular complications (34).

Inevitably, there are certain flaws in this research. Firstly, the sample size was small. Secondly, although it was a long-term assessment to evaluate the risk of DR, the study did not account for the bias caused by patients’ medications as DR progressed. Thirdly, because lighting affects VitD-T3, we could not collect data from patients during a continuous daytime period. As previously said, the risk or the occurrence of DR is not uniform; hence, we need to incorporate more indicators to derive more preventive methods for DR in patients with T2DM.

## Conclusion

This study successfully constructed a nomogram for assessing the risk of DR in patients with T2DM. These findings will guide patients and doctors to develop personalized treatment plans based on these risk factors, eliminate the hazard of DR, and avoid the onset and development of DR.

## Data availability statement

The original contributions presented in the study are included in the article/supplementary material. Further inquiries can be directed to the corresponding authors.

## Ethics statement

Written informed consent was obtained from the individual(s) for the publication of any potentially identifiable images or data included in this article.

## Author contributions

QW, NZ, and HT contributed to the conception and design of the study. QW, NZ, XY, QY, LZ, HZ, YZ, XN, and FJ contributed to data collection, analysis, and interpretation. QW and NZ wrote the article. XL and FJ revised the manuscript. The final article was read and approved by all participants before submission. QW and NZ contributed equally to this work.

## Funding

Thanks to our colleagues at Zunyi Medical College’s Affiliated Hospital for helping us with data collection. This research was funded in part by the Chinese National Science Foundation (82060273) and the Guizhou Technology and Science Program [Qian Ke He Base -ZK (2021)- General 407]. The article’s completion is entirely at the discretion of the funders.

## Conflict of interest

The authors declare that the research was conducted in the absence of any commercial or financial relationships that could be construed as a potential conflict of interest.

## Publisher’s note

All claims expressed in this article are solely those of the authors and do not necessarily represent those of their affiliated organizations, or those of the publisher, the editors and the reviewers. Any product that may be evaluated in this article, or claim that may be made by its manufacturer, is not guaranteed or endorsed by the publisher.
